# Sex and gender as contributors to brain pathophysiology, clinical course, and therapeutic response in multiple sclerosis

**DOI:** 10.3389/fnbeh.2026.1777361

**Published:** 2026-03-10

**Authors:** Stella Panou, Lucia Lucy Privitera

**Affiliations:** Barts and the London School of Medicine, Institute of Health Sciences Education, Queen Mary University of London Malta Campus, Victoria, Malta

**Keywords:** gender differences, multiple sclerosis, neurodegeneration, neuroinflammation, sex differences, treatment

## Abstract

Multiple sclerosis (MS) is a chronic autoimmune demyelinating disorder that affects the brain and spinal cord. MS is characterized by different neurological and cognitive impairments. Several lines of evidence suggest sex-based differences in the incidence, clinical course and pathophysiology of the disease. Epidemiological data show that women are three times more likely to suffer from MS compared to men and tend to present symptoms earlier. Other evidence indicates that men experience more aggressive forms of MS and women respond better to certain disease-modifying drugs. In this mini review, we summarized recent findings on biological, hormonal and psychological factors underpinning these differences, with reproductive stage being recognized as a key variable to be considered in drug safety and efficacy. Beyond biology, sex and gender influence perception of the disease, quality of life and management. Recognizing sex and gender as important factors in MS supports the move toward precision medicine, leading to care that is not only more effective but also more equitable.

## 1 Introduction

Multiple sclerosis is a chronic autoimmune inflammatory demyelinating condition of the central nervous system (CNS). It causes neurological debility in young people, largely impairing quality of life, frequently resulting in long-term disability and an extensive socioeconomic burden on healthcare systems globally (Oh et al., 2018). An estimated 2.8 million people were affected by MS in 2020, rising to 2.9 million by 2024 (Mapping multiple sclerosis around the world key epidemiology findings, 2020). While the reasons for this sex-based difference in MS risk remain unclear, it is likely influenced by genetic and hormonal variances, as well as differences in environmental and social exposures. Current approaches in the treatment of MS are centered on symptom relief, controlling disease progression, and decreasing the number of relapses. Treatment results can differ greatly in patients, suggesting the need for more individualized, gender-specific management. This review aims to examine the impact of biological sex on disease progression and therapeutic outcomes while also discussing how gender related factors may shape disease experience, management, and quality of life. In this context, sex refers to biological attributes, including chromosomal complement, gonadal structure, and endogenous hormone profiles, whereas gender refers to socially constructed roles and identities that shape lived experience, health behaviors, and access to care.

## 2 Sex based pathophysiology

**FIGURE 1:**
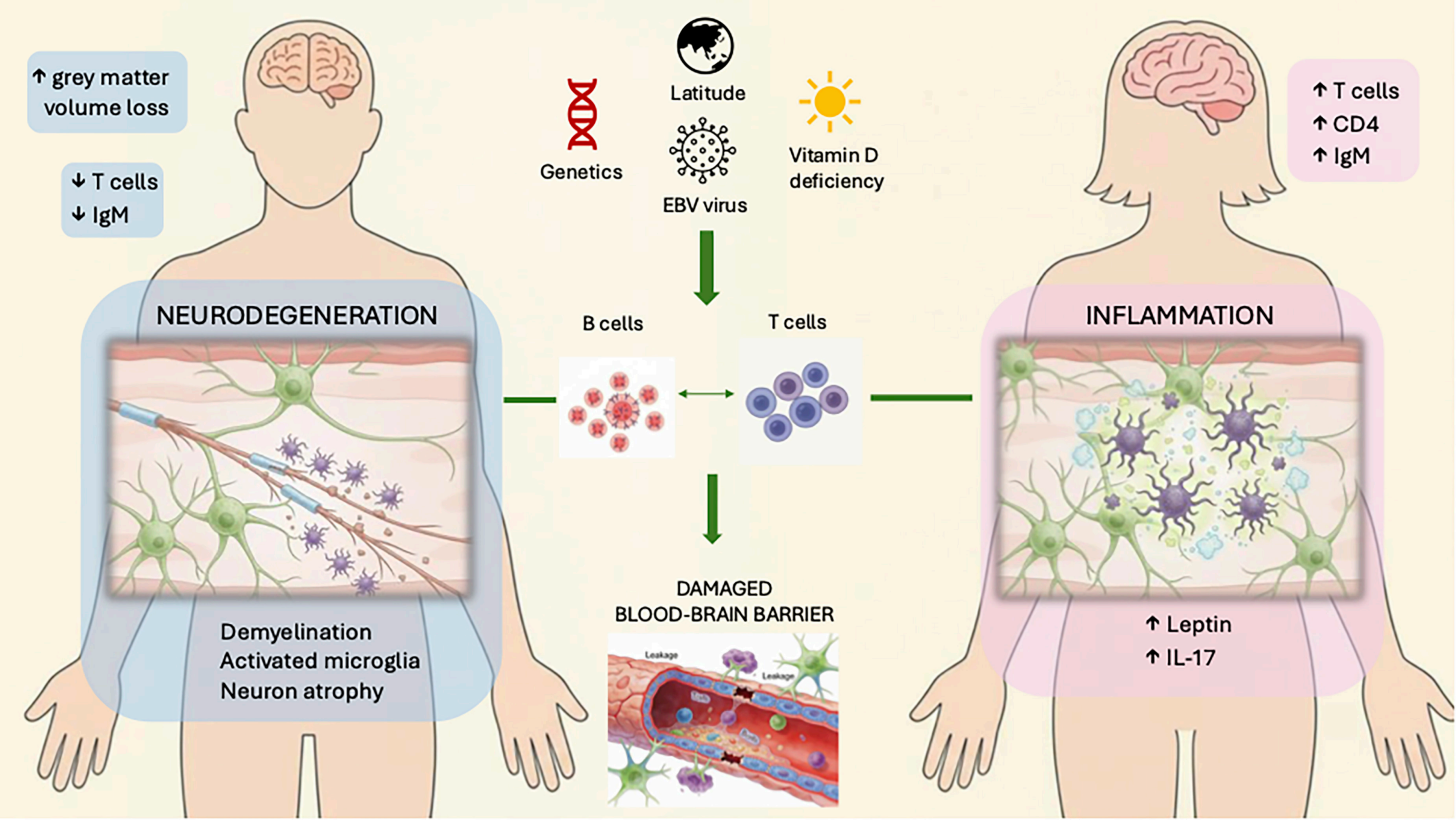
Pathophysiology of multiple sclerosis in males and females. Genetics, EBV virus exposure and the environment (vitamin D deficiency, high latitude) are factors that influence the pathophysiology of MS. T-cell-mediated inflammation which is more pronounced in females, causes neuronal damage and disturbs the BBB integrity. Pro-inflammatory cytokines (IL-17) and higher levels of CD4+ Th1 and Th17 cells as well as leptin further promote inflammation. In males neurodegeneration is the driving pathologic process leading to greater loss of gray matter volume. B-cells also produce antibodies against myelin contributing to demyelination.

T and B lymphocytes play critical roles in MS, driving lesion formation and relapses (Figure 1; Ward and Goldman, 2022). Inflammation compromises blood-brain barrier (BBB) integrity, increasing permeability and perpetuating immune cell infiltration (Liu et al., 2022). Demyelination, the disease hallmark, manifests as plaques damaging myelin, oligodendrocytes (OLs) and axons (Schirmer et al., 2019).

Sex-based differences in the CNS and immune system are influenced by genetic and hormonal factors, significantly impacting MS (Ortona et al., 2016). Physiologically, males have larger brain size, more natural killer cells and lymphocytes, while women have a higher percentage of gray matter (Kanaan et al., 2012; Ingalhalikar et al., 2014).

Hormones like estrogen enhance serotonin function and immune responses, leading to better outcomes in infections but higher rates of autoimmune disorders (Ortona et al., 2016). Nevertheless, men have fewer T-cells compared to women, leading to weaker innate immune responses, although B-cell levels do not differ by sex (Klein and Flanagan, 2016). Women often exhibit higher leptin levels (linked to inflammation), while men show testosterone-related neuroprotection (Klein and Flanagan, 2016). While the exact mechanisms behind these sex differences are not fully understood, sex steroids appear to influence many of these variations (Ryan and Mills, 2022).

Notably, sex-dependent differences in oligodendrocyte precursor cell (OPC) variability have been observed, with females expressing more OPC proliferation and migration. This highlights the potential of sex-specific gene expression in OPCs as a target for new therapies (Kato, 2025). Concluding, limited knowledge of CNS, MS and pathophysiological differences between males and females, restricts the potential of advancements in MS treatment approaches. This mini-review examines current evidence on sex-based pathophysiology, clinical presentation, and treatment response in MS, while also addressing the role of gendered experiences in disease outcomes. Key controversies, knowledge gaps, and implications for precision and hormone-based therapeutic approaches are discussed.

## 3 Sex and gender differences in clinical presentations and prognosis

### 3.1 Classification of MS

MS has three primary patterns of disease course: relapsing-remitting (RRMS), secondary progressive (SPMS) and primary progressive (PPMS). It is well established that females are disproportionately affected by MS, particularly the RRMS subtype. Their disease course is typically more inflammatory, with higher relapse rates linked with elevated levels of pro-inflammatory cytokines (Greer and McCombe, 2011). Conversely, males are less frequently diagnosed with MS but are more likely to present with progressive disease subtypes, such as PPMS or SPMS, which are driven by neurodegeneration rather than inflammation (Koch et al., 2010; Bergamaschi, 2007). Females generally have a lesser prevalence of PPMS, but there is limited research examining sex specific factors influencing disability progression in PPMS (Greer and McCombe, 2011; Bergamaschi, 2007). Testosterone in men appears to exert neuroprotective effects by reducing demyelination and promoting remyelination, which may partially explain the delayed onset of disability in younger men, compared to older men, who experience a decline in testosterone levels correlating with increased disease progression (Greer and McCombe, 2011).

### 3.2 Sex differences in MS presentation

When comparing male and female MS patients, cognitive deficits, reduced functional connectivity and lower network efficiency are observed in both groups (Kanaan et al., 2012; Ingalhalikar et al., 2014). Additionally, male MS patients tend to show more pronounced changes in white matter diffusion, which correlate with greater cognitive impact (Fazekas et al., 2009; Costello et al., 2012). Men experience greater retinal nerve fiber thinning post-optic neuritis (Costello et al., 2012). They also exhibit more severe motor and cerebellar symptoms and a faster rate of brain atrophy, contributing to greater overall disability over time (Bergamaschi, 2007).

The gold standard diagnostic tool to detect periventricular and intracortical lesions in MS is magnetic resonance imaging (MRI) of the brain and spinal cord. Sex-based differences in MRI findings in MS remain debated due to limited sample sizes. Men may have fewer contrast-enhancing lesions but more gray matter volume loss, T1 hypointense lesions and spinal cord axon loss, indicative of irreversible axonal damage and neurodegeneration (Leavitt et al., 2024). Women exhibit more gadolinium-enhancing lesions, reflecting active inflammation consistent with their inflammatory disease course.

A cross-sectional MRI study comparing male and female MS patients with matched controls revealed more atrophy in men’s thalamus, putamen, precuneus and cortical gray matter (Voskuhl et al., 2020). Despite its robust imaging techniques, the study lacked participant detail and confounder analysis.

### 3.3 Sex differences in treatment response and management

Various disease-modifying drugs (DMDs) are used for MS, aiming to reduce the relapse frequency and slow the disease progression. There are limited active comparator trials of different DMDs, and even less studies have explored the impact of sex response to immunotherapy (Jakimovski et al., 2024). Most of the findings are difficult to interpret due to the differences in clinical course and outcomes between men and women. A large cohort study involving 2,570 interferon-beta treated RRMS patients, followed for up to 7 years, suggests that male sex may reduce the risk of a first relapse in patients with a low pre-treatment relapse history (Trojano et al., 2009). Conversely, males who started treatment at a young age but experienced treatment delays, were associated with an increased risk of disease progression. While women generally experience higher relapse rates and inflammation-driven disease activity, they often show more pronounced responses to lower efficacy therapies (Greer and McCombe, 2011). In contrast, men’s slower immune recovery may necessitate high-efficacy therapies to address progressive disease pathways effectively. A randomized placebo-controlled study examined the interferon-beta-1a outcomes on SPMS, focusing on disability progression and relapse rates (SPECTRIMS Study Group, 2001). While no substantial benefit was detected in overall disability progression, exploratory analyses revealed notable gender-specific differences. Women showed a delayed progression of disability and greater improvement in relapse-related outcomes compared to men, who did not experience significant benefits (SPECTRIMS Study Group, 2001). Strengths of this study include rigorous randomization, long-term follow-up and comprehensive subgroup analyses. Limitations include the *post hoc* nature of some analyses and reliance on retrospective data for covariates, which may affect the robustness of conclusions. Another study highlighted that females were more likely to discontinue glatiramer acetate treatment due to intolerable side effects, although no major differences in medication effectiveness was noted (Baiamonte et al., 2025). Additionally, Natalizumab has been shown to reduce relapse rates more effectively in women, likely due to their higher baseline inflammatory activity (Spelman et al., 2015). Conversely, men, who are more prone to progressive forms of MS characterized by neurodegeneration, may benefit more from therapies like siponimod that target neurodegenerative pathways (Scott, 2020).

These findings highlight the influence of biological and immunological sex differences in treatment response and emphasize the need for personalized treatment strategies (Table 1). The study emphasizes the significance of incorporating sex-stratified analyses in clinical trials and MS management, particularly as females respond better to interferon-beta-1a therapy, while alternative methods should be considered for men.

**TABLE 1:** Key differences between females and males with multiple sclerosis.

Category	Females	Males
Prevalence	Higher (3:1)	Lower
Onset	Earlier	Later
Subtype of MS	Mainly RRMS	Mainly SPMS/PPMS
Symptoms	Less cognitive impact	More motor and cerebellar symptoms
Relapse rate	Higher	Lower
Psychosocial	Depression/anxiety more common	Stigma more common
Response to treatment	Better with interferon-β and Natalizumab	Better with high efficacy DMDs and glatiramer
Structural changes	More gadolinium enhancing lesions	Greater gray matter volume loss
Other diagnostic markers	Higher B cell response and oligoclonal band positivity	Higher serum neurofilament light chain levels
Progression	Slower	Faster and more severe

### 3.4 Precision and hormone-based approaches

Precision medicine is a transformative approach to managing MS (Lorefice and Lugaresi, 2025). MS is highly heterogeneous, with variability in symptoms, progression and treatment responses. Current DMDs target peripheral immune mechanisms, but often overlook CNS inflammation and neurodegenerative processes, crucial in progressive MS (Lorefice and Lugaresi, 2025). Advanced imaging differentiates lesion types showing active inflammation or irreversible damage, aiding disease severity stratification (Comabella et al., 2016). Antibody-antigen profiles, such as cerebrospinal fluid (CSF) oligoclonal bands, reveal B-cell-mediated autoimmunity, while serum anti-MOG antibodies identify atypical MS phenotypes. Pharmacogenomic studies link HLA-DRB1*15:01 to MS susceptibility and polymorphisms in CYP2C9 and CYP2J2 to drug metabolism variations (Lorefice et al., 2023).

Biomarkers reveal sex differences in disease monitoring. Serum neurofilament light chain (sNfL), a marker of neuroaxonal damage, rises in both sexes but more in men, reflecting greater neurodegeneration (Cross et al., 2024). Women exhibit stronger B-cell responses, including higher CSF oligoclonal band positivity (Cross et al., 2024). Serum and CSF cholesterol changes linked to NfL show sex-specific patterns (Moles et al., 2025). Six blood proteins consistently differ in PPMS and RRMS (Brichette-Mieg et al., 2025). While these biomarkers could guide personalized treatment, many still lack standardized sex-specific reference ranges.

Matching patients to therapies targeting specific pathological mechanisms enhances quality of life and optimizes resource use. By addressing MS’s biological complexity, precision medicine improves outcomes and reduces costs (Hansen and Okuda, 2018). Future directions include immune profiling for targeted autoimmune management, patient-optimized cellular therapies, like autologous hematopoietic stem cell transplantation, and pharmacogenomics, to minimize adverse effects (AEs) and enhance outcomes (Gourraud et al., 2014).

Sex hormones have a central role in this sex bias, with estrogens and androgens being stimulators of autoimmunity and having protective properties (Ortona et al., 2016; Kaufhold et al., 2025).

Estrogens may have therapeutic benefits for MS by immune response modulation and neuroprotection (Gold and Voskuhl, 2009). However, the safety of estrogen must be carefully evaluated in both genders. Estriol is considered the safest for women during menopause, though it may cause side effects like gynecomastia in men. A preliminary trial administering oral estriol daily in women showed positive immunological effects, which were linked to a reduction in lesions on MRI (MacKenzie-Graham et al., 2012). There is also growing evidence through animal studies suggesting that estrogens could preserve gray matter (MacKenzie-Graham et al., 2012). This discovery may open new avenues for research on hormonal therapies in MS.

Both men and women suffering from MS have lower levels of testosterone when compared to healthy persons (Murgia et al., 2022). Testosterone has been shown to have anti-inflammatory and neuroprotective effects in studies of animal models (Chitnis et al., 2018). Similarly, while testosterone appears to offer neuroprotection for motor neurons, it may exacerbate excitotoxicity in OLs, potentially leading to detrimental effects (Caruso et al., 2004).

The levels of estrogens and progesterone rise during pregnancy, then decrease sharply after childbirth. This could be involved in immune remodeling. Combining DMTs with estrogen or in combination with a progestin receptor modulator that promotes myelin could offer additional therapeutic benefits (Murgia et al., 2022). The neuroprotective role of progesterone remains controversial. Animal studies suggest that progesterone has some neuroprotective effects, but it could also antagonize the beneficial effects of estrogens (Carroll et al., 2008).

Prolactin is known to enhance the number and differentiation of OPCs into mature OLs, which may partially explain the protective effects of breastfeeding in MS (Gregg, 2009). The prolactin receptor is found on immune cells such as peripheral T and B lymphocytes, thymocytes and dendritic cells. Prolactin could therefore modify the responses of CD4+ T-cells by acting on T-bet expression (Tomio et al., 2008).

Ultimately, the role of sex hormones and their application for treatment is multi-faceted and needs more assessment. Although preliminary data are promising, the mechanisms by which sex hormones modulate MS pathophysiology remain not fully understood and hormone-based therapies for MS remain experimental. Larger randomized controlled trials are therefore required to establish safety, efficacy, and sex-specific dosing.

## 4 The role of gendered experience in multiple sclerosis outcomes

### 4.1 Quality of life

MS significantly impacts mental health, causing emotional distress, social isolation and lower quality of life (QoL). Anxiety, depression and stress are common but alleviated by psychological support (Batista et al., 2022). Active coping, such as planning and positive reframing, improves QoL. Fatigue and cognitive issues early in MS reduce work productivity, mobility and mood (Kobelt et al., 2017). Results from a recent analysis revealed that postmenopausal women had worse progression of disability unrelated to relapses, compared to men (Foschi et al., 2025). A qualitative study showed self-care preserves autonomy; however, many women report difficulty balancing disease management with socially ascribed household and caregiving responsibilities, reflecting gendered role expectations (Bravo-González and Álvarez-Roldán, 2019). Emotional support is critical, especially for men facing who may face stigma related to traditional gender norms. Integrating caregivers into MS care plans and addressing gender-specific needs can improve long-term care. Improved healthcare access and gender-specific education are vital for tackling MS’s public health challenges (Boorgu et al., 2022).

Fatigue is known as the greatest issue experienced in MS. In the acute period, high dose corticosteroids are given (Lamb, 2022). However, repeated use, is associated with gastrointestinal upset, osteoporosis (especially in postmenopausal women), hypertension, hyperglycemia, mood disturbances and insomnia. Studies have failed to examine the possible sex-based differences in pharmacokinetics and side effect profile, underlining the need for further research in this area.

Compared to the general population, MS patients are more likely to have comorbid disorders (Marrie et al., 2023). Notable was the greater incidence of depression, anxiety and bipolar disorder in women (Marrie et al., 2023). Identifying appropriate care models can help improve the integration of comorbidity management into MS care, which is crucial.

### 4.2 Fertility and contraception

Current consensus suggests that MS does not generally affect fertility in men (Coyle, 2021). However, a study using data from the Danish MS Registry and National *In Vitro* Fertilization registry found an association between male infertility and MS (Coyle, 2021). This suggests potential links to hypogonadism or an immune-mediated component. A recent study indicated that lower levels of anti-Müllerian hormone, a marker of ovarian aging, were linked with increased disability and greater gray matter loss in females with MS, independent of age and disease length (Graves et al., 2018).

For women with MS, managing fertility plans is crucial, requiring the use of effective contraception. While some studies suggest an association between oral contraceptives and a higher MS activity, others indicate that certain progesterone-based contraceptives, such as medroxyprogesterone acetate, modulate microglial activity, thus reducing demyelination symptoms (Mohammadi et al., 2021). These results underline the value of individualized contraceptive choices based on biological sex, disease course, treatment regimen, and personal preferences.

### 4.3 Pregnancy and breastfeeding

Pregnancy is a significant consideration in MS since it typically affects young women of childbearing age. Generally, MS has minimal to no effect on fertility, pregnancy, or fetal health (Krysko et al., 2023). MS during pregnancy is not considered high-risk and there is no evidence of paternal MS affecting pregnancy outcomes. MS disease activity typically reduces during pregnancy, especially in the third trimester; however, severe relapses can occur, particularly in women who discontinue potent DMDs (Krysko et al., 2023). Relapse rates tend to rise postpartum, especially in the first three months, before reverting to pre-pregnancy levels (Coyle, 2016).

During pregnancy, the T-cell repertoire becomes more evenly distributed, with increased regulatory T-cells inhibiting disease progression (Badam et al., 2022). Notably, higher IL-10 levels are observed during that time, which have immunosuppressive effects. These changes are driven by fluctuating hormone levels. HCG modifies dendritic cell activity, reduces T-cell activation and suppresses cytokine production (Murgia et al., 2022). Estrogen suppresses pathogenic Th17 cells, promotes anti-inflammatory cytokine production and enhances Treg proliferation, while progesterone induces T cell death thus reducing MS activity (Iannello et al., 2019). Physiological adaptations also protect the myelin sheath. Pregnancy-related neurosteroids and placental factors may further support myelin repair and neuroprotection (Kalakh and Mouihate, 2019).

In managing MS during pregnancy, minimizing unnecessary medication use is key. Injectable medications like glatiramer acetate and interferon-beta had no teratogenic effects and a good safety profile. Other DMDs, however, lack sufficient human exposure data to draw definitive conclusions (Nytrova and Dolezal, 2022). Natalizumab carries a risk of significant disease rebound during pregnancy (Coyle, 2025). Oral S1P receptor modulators also pose a relapse risk and are avoided in pregnancy due to animal studies indicating growth-related toxicity (Coyle, 2025).

Breastfeeding is generally considered safe during treatment with steroids, glatiramer acetate, interferon-beta and monoclonal antibodies (Krysko et al., 2023). Although IgG antibodies can pass into breast milk, they are typically broken down in the infant’s gastrointestinal tract. As more data becomes available, updates will be essential to manage MS during pregnancy safely.

Beyond pregnancy and breastfeeding, menopause represents another hormonally vulnerable period in women with MS, during which symptoms can worsen (Midaglia et al., 2022). However, the long-term effects of menopause remains unclear and understudied (Midaglia et al., 2022).

## 5 Discussion

This review has examined how biological sex and gendered experiences independently and interactively shape multiple sclerosis prognosis. While women are more frequently affected by MS, men tend to face a more aggressive disease course with earlier disability advancement, greater cognitive decline and more severe motor and cerebellar symptoms. The shift to a progressive course in MS reflects a transition from localized acute injury to widespread inflammation and neurodegeneration, along with compromised compensatory mechanisms such as remyelination and neuroplasticity. This suggests viewing MS as a spectrum influenced by overlapping pathological and reparative processes.

Even though increasing recognition of sex-biased molecular regulation across diseases, the principal mechanisms driving these differences in MS remain incompletely understood. Sex-specific responses to therapies remain inadequately addressed. In contrast, gender-related determinants such as healthcare access, caregiving burden, and treatment adherence remain underexplored in clinical trial design. Women, generally respond better to first-line anti-inflammatory therapies, whereas men may derive more benefit from neurodegenerative-targeting treatments. However, only recent clinical trials have stratified outcomes according to sex and analyses on drug safety profiles have only incorporated sex as a confounder rather than a key determinant of the hypothesis.

Emerging biological evidence, not fully covered in this mini review, suggest that baseline sex-specific gene expression and epigenetic regulation may shape disease susceptibility and progression. Sex-differential autosomal gene expression regulated by estrogen and androgen response elements has been demonstrated in healthy gastric tissue (Mohammadi et al., 2019), indicating that transcriptional dimorphism is not disease-specific. Transcriptomic analyses in thyroid cancer has shown that sex-specific networks and differential expression of X- and Y-linked histone demethylases KDM5C, KDM5D and KDM6A (Shobab et al., 2024). These findings suggest that chromosomal complement and epigenetic regulation, alongside hormones, may drive sexual dimorphism. In MS, sex may thus act as a molecular variable, with pre-existing sex-dependent regulatory pathways as potentially shaping immune responses, neurodegeneration, and repair.

Despite encouraging treatment developments, critical gaps remain in understanding how sex and gender influence MS progression and clinical outcomes. Clinical trials often lack adequate sex-specific analyses, limiting the ability to tailor treatments effectively. Given the observed sex differences in disease pathology such as stronger B-cell-mediated responses in women and greater neurodegenerative burden in men, precision medicine approaches are vital for individualized care. Integrating sex and gender-specific considerations in MS is crucial for advancing disease management strategies. As a narrative mini-review, this work does not include quantitative meta-analysis; thus, potential selection bias and heterogeneity should be considered. Future systematic reviews may better quantify these effects.
